# Metric Anatomy of Pain: A Critical Analysis of Assessment Tools in Algological Practice

**DOI:** 10.7759/cureus.112532

**Published:** 2026-07-12

**Authors:** Juan J Valero Quintero, Héctor Aceituno, Luisana Maldonado-De Santiago, Jennifer Vela-Poveda, José Vásconez-Fabre

**Affiliations:** 1 Neurosurgery, Central University of Venezuela, Caracas, VEN; 2 Neurosurgery, Hospital San Juan de Dios de Curicó, Curicó, CHL; 3 General Medicine, Servicio de Alta Resolutividad (SAR) Bombero Garrido, Curicó, CHL; 4 General Medicine, Centro de Salud Familiar (CESFAM) Oriente, San Fernando, CHL; 5 Neurosurgery, Clínica Miguel Claro, Santiago, CHL

**Keywords:** behavioral pain scales, biopsychosocial model, chronic pain, cosmin, cross-cultural adaptation, nociplastic pain, pain assessment, pain measurement, patient-reported outcome measures, psychometrics

## Abstract

Pain assessment conditions the quality of the therapeutic process in a context reshaped by the 2020 International Association for the Study of Pain (IASP) definitional update, the 2017 introduction of the nociplastic mechanistic descriptor, and the recognition of chronic primary pain as an autonomous nosological entity in the International Classification of Diseases, 11th Revision (ICD-11). In this scenario, we conducted a structured narrative review of hybrid architecture, conceived as an instrument mapping review with the deliberate incorporation of elements proper to systematic reviews of measurement instruments, following the Scale for the Assessment of Narrative Review Articles (SANRA) and the principles of the Preferred Reporting Items for Systematic Reviews and Meta-Analyses extension for Scoping Reviews (PRISMA-ScR). A systematized search was performed across seven databases (PubMed/MEDLINE, Cochrane Library, Embase, Scopus, PsycINFO, CINAHL, and BVS-LILACS) from January 2000 to February 2026, complemented by hand-searching the guidelines of the main international algological societies (IASP, Initiative on Methods, Measurement, and Pain Assessment in Clinical Trials {IMMPACT}, American Geriatrics Society {AGS}, European Pain Federation (EFIC), Society of Critical Care Medicine {SCCM}/Pain, Agitation/Sedation, Delirium, Immobility, Sleep {PADIS}, Institute for Clinical Systems Improvement {ICSI}). The research question was structured according to the COnsensus-based Standards for the selection of health Measurement INstruments guidelines for patient-reported outcome measure (COSMIN-PROM) framework, and the 79 included studies were critically appraised using a principal evaluative core (COSMIN Risk of Bias, A MeaSurement Tool to Assess systematic Reviews, version 2 {AMSTAR-2}, and Quality Assessment of Diagnostic Accuracy Studies, version 2 {QUADAS-2}), complemented by RoB 2, ROBINS-I, and AGREE II for the corresponding subsets, with additional calculation of derived diagnostic indices (positive and negative likelihood ratios, diagnostic odds ratio, and Youden index) for screening scales. Instruments were organized in a four-axis operational taxonomy comprising unidimensional intensity scales, multidimensional scales, neuropathic pain-specific scales, pediatric scales, observational scales for non-communicative critically ill patients, observational scales for cognitive impairment and dementia, functional and quality-of-life scales, and psychological scales. The majority of the psychometric studies included (63% per COSMIN Risk of Bias) exhibited low risk of bias, while the principal psychometric deficits concentrated in the limited discriminant validity of neuropathic screening scales vs. nociplastic pain, the insufficient responsiveness evidence in observational geriatric scales, and the heterogeneity of Spanish and Portuguese cross-cultural adaptations. No universal scale exists; optimal selection depends on clinical context, age, cognitive status, mechanistic descriptor, and evaluation objectives. The multimodal integration of unidimensional instruments with functional, cognitive, and behavioral scales operationalizes the biopsychosocial approach that constitutes the contemporary standard for comprehensive chronic pain assessment. The COSMIN-compliant adaptation and revalidation of key instruments in Ibero-American populations constitutes a translational research priority.

## Introduction and background

Pain is one of the most frequent reasons for medical consultation worldwide and represents a complex biopsychosocial phenomenon whose correct assessment decisively conditions the quality of the therapeutic process. In its 2020 revision, the International Association for the Study of Pain (IASP) defines it as "an unpleasant sensory and emotional experience associated with, or resembling that associated with, actual or potential tissue damage" [1]. The substantive contribution of this revision lies less in the definition itself than in its accompanying explanatory notes, which clarify that pain and nociception (the neural detection of a noxious stimulus) are distinct phenomena and that an inability to communicate does not negate the possibility of experiencing pain [1]. These clarifications carry a direct clinical consequence as follows: they make pain assessment a technical competence in its own right and create an explicit obligation to evaluate populations that cannot self-report, including infants, older adults with cognitive impairment, critically ill patients, and individuals with communicative disability.

The scale of the problem is considerable. Using Global Burden of Disease methodology, contemporary estimates place the worldwide prevalence of chronic pain in adults between 27% and 38%, depending on the operational definition employed [2]. Its economic impact is comparably large - in the United States, the direct and indirect costs of chronic pain have been estimated at approximately 560-635 billion dollars annually, close to 2.5% of gross domestic product and exceeding the combined costs of cardiovascular disease, cancer, and diabetes [2]. Reflecting this burden, the 11th revision of the International Classification of Diseases (ICD-11), introduced in 2022, introduced a dedicated category for chronic primary pain (code MG30), formally recognizing chronic pain as a disease in its own right rather than merely a symptom of an underlying condition [3]. This reclassification reshapes how chronic pain is diagnosed and managed.

The measurement of pain carries an instructive cautionary precedent. The 1996 designation of pain as the "fifth vital sign," later endorsed to encourage routine assessment, raised clinical awareness but proved counterproductive in practice, as pressure to lower numerical scores has been epidemiologically linked to excessive opioid prescription [4]. The lesson is that quantifying intensity is necessary but not sufficient - a single number must be integrated into a broader framework that also captures function and psychosocial impact.

The choice of assessment instrument is therefore far from trivial, as it determines the sensitivity of pain detection, the ability to monitor treatment response, and the comparability of results across studies. The Initiative on Methods, Measurement, and Pain Assessment in Clinical Trials (IMMPACT) has established a widely accepted standard recommending that chronic pain be evaluated across multiple domains - intensity, physical function, emotional function, global improvement, adverse events, and patient disposition [5]. Selecting rationally among the available scales requires knowing their psychometric properties (whether an instrument measures consistently and detects real change), the populations in which they were validated, their operational feasibility, and which mechanism of pain they are suited to - nociceptive, neuropathic, or nociplastic - the last referring to pain arising from altered central pain processing without identifiable tissue or nerve damage and recognized by IASP as a third independent descriptor in 2017 [6,7]. With more than 50 validated scales now in use, selecting the appropriate one has itself become a clinical challenge. In practice, these instruments serve distinct purposes as follows: unidimensional scales rapidly quantify intensity, multidimensional questionnaires capture the sensory, emotional, and functional burden of pain, screening tools flag a probable neuropathic component, and observational scales infer pain in patients who cannot report it - each suited to a particular clinical situation rather than universally interchangeable.

Previous reviews have addressed parts of this landscape, focusing for instance on unidimensional scales in adults or on comparative properties in chronic low back pain [8], yet important gaps remain as follows: few have integrated recent conceptual developments such as nociplastic pain, the ICD-11 MG30 category, or emerging artificial intelligence tools for facial pain recognition; few have applied formal psychometric appraisal criteria to the algological domain; and few have addressed the Ibero-American context, where culturally adapted Spanish-language versions are unevenly available and chronic pain prevalence reportedly ranges between 25% and 42% across Chilean, Mexican, Colombian, and Brazilian studies [9]. Against this background, the present review pursues a defined objective within a defined scope as follows: to identify which instruments are available for assessing pain across different care settings, which psychometric properties support them, and how they should be selected according to clinical context, age, cognitive status, pain mechanism, and assessment objectives. Its aim is to provide the clinician with a conceptual and operational framework of high translational value, aligned with international methodological standards and attentive to the particularities of the Ibero-American setting.

## Review

Methodological framework

Design, Guidelines, and Research Question

A structured narrative review of hybrid character was performed, conceived as an instrument-mapping review incorporating elements proper to systematic reviews of measurement instruments. This dual architecture preserves the interpretive, translational orientation of a narrative review while ensuring the evaluative rigor that the psychometric nature of the object of study demands. The narrative-interpretive component followed the recommendations of the Scale for the Assessment of Narrative Review Articles (SANRA) [10], and the identification, selection, and characterization of instruments followed the principles of Preferred Reporting Items for Systematic Reviews and Meta-Analyses extension for Scoping Reviews (PRISMA-ScR), as the review meets the operational characteristics of an instrument scoping review as follows: broad question, multi-database systematized search, explicit eligibility criteria, and structured mapping [11]. Critical appraisal followed the COSMIN guideline for systematic reviews of patient-reported outcome measures (PROMs) [12]. A systematic review stricto sensu was deliberately not adopted because the primary objective is interpretive synthesis oriented to the clinician rather than aggregated effect quantification; the clinical, population, and psychometric heterogeneity of the included studies renders meta-analysis unfeasible; and the inclusion of instruments from multiple taxonomic categories responds to a logic of comprehensive mapping rather than a focused clinical question. International Prospective Register of Systematic Reviews (PROSPERO) registration was therefore not applicable; the working protocol was prospectively elaborated and agreed upon before search initiation and is available from the corresponding author.

The orienting question was structured according to the COSMIN-PROM framework [12]. Population - adult, pediatric, geriatric with cognitive impairment, and critically ill non-communicative patients, evaluated for acute or chronic pain in any care setting; construct - pain experience in its dimensions of intensity, sensory quality, functional impact, affective-cognitive component, and underlying mechanism (nociceptive, neuropathic, nociplastic); instruments - validated self-report and observational scales and questionnaires; measurement properties - validity (content, criterion, construct, cross-cultural), reliability (internal consistency, test-retest, interrater), responsiveness, minimal clinically important difference (MCID), and operational feasibility [6].

Search, Eligibility, and Critical Appraisal

The bibliographic search was performed in PubMed/MEDLINE, Cochrane Library, Embase, Scopus, PsycINFO, CINAHL, and Virtual Health Library - Latin American and Caribbean Health Sciences Literature (BVS-LILACS), from January 1, 2000, to February 28, 2026, with emphasis on the last decade. Foundational studies prior to 2000 were retained when constituting the original publications of the evaluated scales. Descriptors were drawn from the Medical Subject Headings (MeSH) and Health Sciences Descriptors (DeCS) thesauri (including pain measurement, pain assessment, the principal named scales, and the psychometric-property terms) combined through Boolean operators. Hand-searching was performed in the references of identified systematic reviews and in the clinical practice guidelines of the IASP, IMMPACT, American Geriatrics Society (AGS), European Pain Federation (EFIC), Society of Critical Care Medicine (SCCM) (Pain, Agitation/Sedation, Delirium, Immobility, Sleep {PADIS} guideline), and Institute for Clinical Systems Improvement (ICSI).

Included studies were as follows: original psychometric validation studies; COnsensus-based Standards for the selection of health Measurement INstruments (COSMIN)-based systematic reviews and meta-analyses [7,12]; cross-cultural adaptation studies; prospective observational studies and randomized controlled trials providing data on validity, reliability, responsiveness, or MCID; formal guidelines and consensus of the organizations cited; and head-to-head comparative studies. Excluded were letters without primary data, expert opinions unsupported by evidence (formal guidelines excepted), studies with sample sizes below COSMIN thresholds (n ≥ 100 for confirmatory factor analysis; n ≥ 50 for test-retest, internal consistency, and responsiveness; n ≥ 30 for qualitative content validity), duplicates, and publications in languages other than Spanish, English, French, and Portuguese-French being retained for the relevance of the Francophone school in neuropathic (DN4, Neuropathic Pain Symptom Inventory {NPSI}) and geriatric (DOLOPLUS-2) scales [7]. Selection proceeded through title/abstract screening, full-text review, and final inclusion, with discrepancies resolved by consensus and, when needed, by a third reviewer; bibliographic management was performed in Zotero version 6.0 (Vienna, VA: Corporation for Digital Scholarship).

Critical appraisal was structured at two levels. A principal evaluative core applied the standard tools for formal instrument appraisal as follows: the COSMIN Risk of Bias checklist to studies of psychometric properties [13], A MeaSurement Tool to Assess systematic Reviews, version 2 (AMSTAR-2) to systematic reviews and psychometric meta-analyses, and Quality Assessment of Diagnostic Accuracy Studies, version 2 (QUADAS-2) to the diagnostic-accuracy studies validating neuropathic screening instruments against a clinical reference standard [14]. A complementary level applied the Cochrane risk-of-bias tool for randomized trials (RoB 2) to the included randomized trials (n = 5) and Risk Of Bias In Non-randomized Studies - of Interventions (ROBINS-I) to non-randomized observational studies (n = 8), reported descriptively given their reduced number, and Appraisal of Guidelines for REsearch and Evaluation, version II (AGREE II) to guidelines and consensus (n = 7), reporting the global recommendation rather than a risk-of-bias category [15]. Consolidated Standards of Reporting Trials (CONSORT), STrengthening the Reporting of OBservational studies in Epidemiology (STROBE), STAndards for Reporting of Diagnostic accuracy studies (STARD), and Preferred Reporting Items for Systematic Reviews and Meta-Analyses (PRISMA) were used only as reporting references to judge informational completeness. Appraisal was performed by two independent reviewers, with discrepancies resolved by consensus and, when necessary, a third reviewer.

Evidence Synthesis, Derived Indices, and Psychometric Framework

Synthesis was narrative and structural, organized by an operational taxonomy predefined from previous literature and inductively refined, classifying scales by respondent type, dimensionality, mechanistic descriptor, and target population [6-8,12]. Foundational publications and recent validation studies were treated as complementary rather than competing sources as follows: the original development study was retained as the primary reference for an instrument's structure, scoring, and conceptual basis, while the most recent COSMIN-compliant validations were given precedence whenever they updated estimates of reliability, responsiveness, cross-cultural performance, or diagnostic accuracy, or characterized instrument behavior in populations not represented in the original validation. When psychometric results were contradictory for the same scale, priority was given, in order, to COSMIN-based studies, larger samples, more recent comparable studies, and populations closer to the Ibero-American context.

From the sensitivity (S) and specificity (E) reported in primary diagnostic studies, derived indices of clinical utility were calculated by standard formulas as follows: positive likelihood ratio (LR+) = S/(1-E); negative likelihood ratio (LR-) = (1-S)/E; diagnostic odds ratio (DOR) = LR+/LR-; and Youden J = S + E - 1. For studies reporting intraclass correlation coefficient (ICC) or Pearson's r between instruments, the shared-variance fraction r² was derived (r² = ICC² where applicable). The theoretical combined sensitivity of two tests applied in parallel was estimated as 1 - (1-S₁) (1-S₂), with the observed divergence interpreted as an indicator of partial correlation. All derived calculations are identified as such in the tables and footnotes to distinguish them from primary values. The authors did not participate in the development, validation, or adaptation of any evaluated scale, minimizing instrument-affinity bias.

A valid pain assessment scale must satisfy the psychometric requirements that guarantee clinical and research utility as follows: content, criterion, construct, and cross-cultural validity certify that the instrument measures the pain construct in its relevant dimensions [7,12]; reliability (interrater, test-retest, internal consistency) ensures stability and reproducibility; responsiveness determines the capacity to detect clinically significant change; and the MCID defines the threshold of clinical relevance, ranging for most unidimensional scales between 1.5 and 2 points out of 10, or a 30% reduction [16]. Table 1 synthesizes the psychometric standards applied.

**Table 1:** Psychometric standards applied to evaluated scales. CVI: content validity index; ICC: intraclass correlation coefficient; SRM: standardized response mean; AUC: area under the curve; MCID: minimal clinically important difference

Property	Definition	Acceptable threshold
Content validity	Item adequacy to the construct	Expert judgment (CVI ≥ 0.78)
Construct validity	Correlation with related instruments	r ≥ 0.50 (a priori hypothesis)
Internal consistency	Item homogeneity (Cronbach α)	0.70 ≤ α ≤ 0.95
Test-retest reliability	Temporal stability (ICC)	ICC ≥ 0.70
Interrater reliability	Observer agreement (κ or ICC)	κ ≥ 0.60
Responsiveness	Capacity to detect change (effect size, SRM)	ES ≥ 0.80 (high)
MCID	Clinically significant change	~30% baseline or ≥ 2 points/10
Diagnostic validity	Sens/spec against reference standard	AUC > 0.70 acceptable

Methodological Quality of Included Studies

The systematic search performed in the seven consulted databases (PubMed/MEDLINE, Cochrane Library, Embase, Scopus, PsycINFO, CINAHL, and BVS-LILACS) between January 2000 and February 2026, complemented by hand-searching of cross-references and international clinical practice guidelines, identified approximately 1,240 potentially eligible records. After duplicate removal and title/abstract screening, 187 studies were reviewed in full text. Of these, 79 met eligibility criteria and were included in qualitative synthesis, distributed according to methodological design as follows: 38 psychometric validation studies, 12 diagnostic accuracy studies, nine systematic reviews or meta-analyses, eight prospective observational studies, five randomized controlled trials, and seven clinical practice guidelines or formal consensus. Foundational publications prior to the year 2000, constituting the primary source of evaluated scales, were additionally preserved. The included studies were critically appraised with a principal evaluative core (COSMIN Risk of Bias checklist for psychometric and observational studies, AMSTAR-2 for included systematic reviews, and QUADAS-2 for diagnostic accuracy studies) [13,14], complemented by RoB 2 (randomized trials), ROBINS-I (non-randomized observational studies), and AGREE II as a complementary reference for clinical practice guidelines and formal consensus [15]. The global distribution of included studies according to design and result of critical appraisal is synthesized in Table 2.

**Table 2:** Distribution of included studies by design and result of critical appraisal. Categories reported per each tool's original convention. Percentages omitted for n < 10; no aggregated total across tools (non-comparable metrics). PROM: patient-reported outcome measure

Study design	Tool	Evaluative level	n (total = 79)	Result
Psychometric properties studies (PROM and observational)	COSMIN RoB	Principal core	38	Low risk: 24 (63%), moderate: 11 (29%), high: 3 (8%)
Psychometric systematic reviews/meta-analyses	AMSTAR-2	Principal core	9	High quality: 5 (56%), moderate: 3 (33%), low: 1 (11%)
Diagnostic accuracy studies (neuropathic screening)	QUADAS-2	Principal core	12	Low risk: 7 (58%), moderate: 4 (33%), high: 1 (8%)
Randomized controlled trials	RoB 2	Complementary	5	Low risk: 3, some concerns: 2, high: 0
Non-randomized observational studies	ROBINS-I	Complementary	8	Low: 4, moderate: 3, serious: 1, critical: 0
Clinical practice guidelines and formal consensus	AGREE II	Complementary reference	7	Recommended: 7, recommended with modifications: 0, not recommended: 0

Critical appraisal shows a body of evidence that is globally solid in its psychometric core, with concrete areas of methodological uncertainty. In the principal evaluative core, the majority of psychometric studies analyzed with COSMIN Risk of Bias (24/38; 63%) exhibited low risk of bias, and only 8% (3/38) high risk; in QUADAS-2, 7/12 (58%) of diagnostic accuracy studies obtained low risk; and systematic reviews evaluated with AMSTAR-2 attained high or moderate quality in 89% of cases (8/9). At the complementary evaluative level, randomized controlled trials (n = 5) showed low risk in three and "some concerns" in two per RoB 2; non-randomized observational studies (n = 8) were distributed among low risk (four), moderate (three), and serious (one) per ROBINS-I; and all clinical practice guidelines and formal consensus evaluated (n = 7) were categorized as "recommended" per AGREE II. Studies with high risk of bias in the psychometric domain were non-randomly concentrated in validations with suboptimal sample size for confirmatory factor analysis, absence of a priori construct validity hypotheses, or undescribed missing data handling, defects characteristic of older psychometric literature that should motivate contemporary revalidation of several instruments. These findings are reported descriptively and differentiated by tool, without global aggregation, since the categories of each critical appraisal instrument respond to heterogeneous methodological conventions not admitting a common aggregated metric. Regarding specific psychometric properties of the principal evaluated instruments, most standardized scales (visual analog scale {VAS}, numerical rating scale {NRS}, Brief Pain Inventory {BPI}, Short-Form McGill Pain Questionnaire-2 {SF-MPQ-2}, Pain Assessment in Advanced Dementia {PAINAD}, Critical-Care Pain Observation Tool {CPOT}, Behavioral Pain Scale {BPS}) have evidence of methodological quality sufficient for clinical recommendation per COSMIN thresholds. Areas of greatest methodological uncertainty concentrate in (i) lack of responsiveness studies with prolonged follow-up for geriatric scales (DOLOPLUS-2, Abbey, Mobilization-Observation-Behavior-Intensity-Dementia {MOBID-2}); (ii) heterogeneity of cross-cultural adaptation methods for Spanish versions; and (iii) limited discriminant validity evidence between neuropathic and nociplastic pain in neuropathic screening instruments (DN4, Leeds Assessment of Neuropathic Symptoms and Signs {LANSS}, painDETECT) (Table 3).

**Table 3:** COSMIN critical appraisal of principal instruments: reported psychometric. Global quality - high: all/almost all properties sufficient; moderate: ≥ 1 property insufficient; low: multiple properties without evidence. +: sufficient evidence; ?: insufficient/unreported; n/a: not applicable; unidim: unidimensional; obs: observational; LANSS: Leeds assessment of neuropathic symptoms and signs; ICC: intraclass correlation coefficient; VAS: visual analog scale; NRS: numerical rating scale; FPS-R: Faces Pain Scale-Revised; SF-MPQ: Short-Form McGill Pain Questionnaire; BPI: Brief Pain Inventory; BPI-SF: Brief Pain Inventory-short form; LANSS: Leeds Assessment of Neuropathic Symptoms and Signs; S-LANSS: self-administered LANSS; FLACC: Face, Legs, Activity, Cry, Consolability scale; PIPP-R: Premature Infant Pain Profile-Revised

Instrument	Content validity	Internal consistency	Reliability	Structural validity	Construct validity	Responsiveness	Global quality
VAS	+	n/a (unidim.)	+ (ICC > 0.90)	n/a	+	+	High
NRS	+	n/a (unidim.)	+ (ICC > 0.86)	n/a	+	+	High
FPS-R	+	n/a (unidim.)	+ (ICC > 0.80)	n/a	+ (r = 0.93 VAS)	+	High
SF-MPQ-2	+	+ (α = 0.83-0.95)	+ (ICC > 0.75)	+ (4 factors)	+	+	High
BPI/BPI-SF	+	+ (α = 0.82-0.95)	+	+ (2 factors)	+	+	High
DN4	+	+ (α = 0.82)	+	+ (1 factor)	+ (vs. LANSS)	?	Moderate
LANSS/S-LANSS	+	+	+	+	+ (vs. DN4)	?	Moderate
painDETECT	+	+	+	+	+	?	Moderate
FLACC	+	n/a (obs.)	+ (κ > 0.80)	n/a	+	+	High
PIPP-R	+	n/a (obs.)	+ (ICC > 0.90)	+	+	+	High
BPS	+	n/a (obs.)	+ (κ > 0.80)	+ (3 domains)	+	+	High
CPOT	+	n/a (obs.)	+ (κ = 0.80-0.90)	+ (4 domains)	+	+ (ES = 1.4)	High
PAINAD	+	+ (α = 0.75)	+ (κ > 0.82)	+	+	+	High
DOLOPLUS-2	+	+ (α = 0.75-0.82)	+ (ICC > 0.75)	+ (3 domains)	+	?	Moderate
MOBID-2	+	+ (α = 0.84)	+ (κ = 0.77)	+	+	+	High
ODI	+	+ (α = 0.87)	+ (ICC = 0.90)	+ (1 factor)	+	+ (MCID = 6)	High
WOMAC	+	+ (α = 0.86-0.95)	+ (ICC > 0.85)	+ (3 domains)	+	+	High
SF-36	+	+ (α = 0.70-0.95)	+ (ICC > 0.80)	+ (8 domains)	+	+	High
PCS	+	+ (α = 0.87-0.95)	+ (ICC > 0.75)	+ (3 factors)	+	+	High
TSK	+	+ (α = 0.77-0.86)	+ (ICC > 0.80)	+ (2 factors)	+	+	High

Unidimensional intensity scales

Visual Analog Scale (VAS)

The visual analog scale consists of a straight line with verbally anchored extremes ("no pain" and "worst imaginable pain") on which the patient marks the perceived intensity; the observer quantifies the distance in millimeters [17]. Its essentially linear-parametric behavior allows robust statistical calculations and shows excellent correlation with other intensity scales [8]. The systematic review by Chiarotto et al. confirmed its convergent validity and responsiveness in chronic low back pain [8]. Its main limitations stem from the requirement for visuomotor capacity and the conceptual abstraction implied by equating spatial distance with sensory experience, rendering it inadequate for patients with moderate-to-severe cognitive impairment, children under seven years, or significant visual or motor disability.

Numerical Rating Scale (NRS)

The NRS asks the patient to quantify pain through an integer from 0 to 10. Its operational simplicity makes it the most used scale in clinical practice and the one recommended by IMMPACT as primary intensity measure in chronic pain clinical trials [5]. The multicenter INTERVAL study demonstrated robust concordance with the VAS; nonetheless, the authors warned that scores are not interchangeable at the individual level [18]. The conventionally accepted MCID threshold is a reduction of at least 30% with respect to baseline value [16]. The NRS presents substantial operational advantages as follows: it requires no materials, permits verbal, telephonic, or digital administration, has minimal non-response rates, and is comprehensible to patients with low educational levels.

Verbal Rating Scale (VRS)

The VRS uses ordered verbal descriptors of intensity as follows: absent, mild, moderate, severe, unbearable (with variants of 4-7 levels). Its simplicity makes it suitable for elderly patients, those with low educational level, or with mild cognitive dysfunction. Limitations include limited granularity (insufficient to detect subtle changes), lower responsiveness than VAS or NRS, and dependence on a cultural and linguistic lexicon, which hinders cross-cultural equivalence [19].

Faces Pain Scale-Revised (FPS-R)

The FPS-R presents six stylized faces progressing from a neutral expression to maximum pain, deliberately eliminating smiles and tears that may induce affective bias [20]. It is the scale recommended by IASP for pediatric use from four years onward, and its utility extends to elderly patients with mild cognitive impairment and to cross-cultural contexts. The Wong-Baker FACES Pain Rating Scale, a popular alternative, shares the pictographic philosophy but, by including smile and tear, may inflate low scores due to the affective load of the neutral face [21]. Table 4 comparatively synthesizes the properties of unidimensional scales.

**Table 4:** Unidimensional pain intensity scales - psychometric comparison. r²: shared variance fraction (calculated as intraclass correlation coefficient {ICC 2}); MCID: minimal clinically important difference; Sp. validation: availability of validated Spanish version; VRS: verbal rating scale; NRS: numerical rating scale; VAS: visual analog scale; FPS-R: Faces Pain Scale-Revised; IMMPACT: Initiative on Methods, Measurement, and Pain Assessment in Clinical Trials

Scale	Studies	Range	Target population	Correlation with VAS	MCID	Sp. validation	Reference
VAS	Aitken (1969)	0-100 mm	Cognitively intact adults	Standard	15-20 mm or 30%	Yes	[8,17]
NRS	IMMPACT (rec.)	0-10 points	Adults; IMMPACT standard	ICC = 0.86 (r² = 0.74)	2 points or 30%	Yes	[5,16,18]
VRS	Multiple	4-7 levels	Elderly, low education level	r = 0.60-0.80	One categorical level	Yes	[19]
FPS-R	Hicks et al. (2001)	0-10 points (6 faces)	Children ≥ 4 years; elderly	r = 0.93	2 points	Yes	[20]
Wong-Baker	Wong-Baker 1988	0-10 points (6 faces)	Children ≥ 3 years; emergency	r = 0.89-0.95	2 points	Yes	[21]

Multidimensional scales

McGill Pain Questionnaire (MPQ) and Short Forms

The MPQ constitutes the foundational multidimensional instrument of modern algology [22]. It organizes verbal descriptors into four dimensions (sensory, affective, evaluative, and miscellaneous) distributed across 20 subgroups, generating quantitative indices such as the pain rating index (PRI), present pain intensity (PPI), and number of words chosen (NWC). Its theoretical value is exceptional by integrating the affective-motivational dimension of pain, but its extension and complexity limit its routine clinical applicability. The SF-MPQ reduces the original questionnaire to a brief selection of sensory and affective descriptors, complemented by a VAS and a verbal scale of present intensity [23]. The revised SF-MPQ-2 restructures the instrument into the following four subscales: continuous pain, intermittent pain, neuropathic pain, and affective descriptors [23]. This factorial architecture allows simultaneous characterization of nociceptive and neuropathic components of chronic pain. A systematic review following COSMIN methodology documented elevated internal consistency, satisfactory convergent validity, and responsiveness in musculoskeletal pathologies [24].

*Brief Pain Inventory (BPI) and West Haven-Yale Multidimensional Pain Inventory* (*WHYMPI)*

The BPI evaluates the following three domains: (i) intensity (worst, least, average, current), (ii) location through body diagram, and (iii) interference with seven functional dimensions (general activity, mood, walking ability, work, relations, sleep, and enjoyment of life) [25]. Its operational brevity, robust conceptual foundation, and adoption by IMMPACT as the preferred measure of functional interference in chronic pain have consolidated it as the most used multidimensional instrument in research, with psychometric properties confirmed in a COSMIN systematic review [5,24]. The West Haven-Yale Multidimensional Pain Inventory (WHYMPI) explores 12 dimensions of chronic pain impact, grouped in the following three sections: subjective experience, perception of significant environmental responses, and participation in activities [26]. Its cognitive-behavioral orientation makes it especially useful in multidisciplinary chronic pain units, although its extension limits its routine use. Table 5 synthesizes multidimensional scales.

**Table 5:** Multidimensional pain scales - structural and psychometric comparison. Application time is estimated according to primary literature. PPI: present pain intensity; Sp. val.: Spanish validation; WHYMPI: West Haven-Yale Multidimensional Pain Inventory; BPI: Brief Pain Inventory; BPI-SF: Brief Pain Inventory-short form; SF-MPQ: Short-Form McGill Pain Questionnaire; MPQ: McGill Pain Questionnaire; VAS: visual analog scale; PPI: Present Pain Intensity

Scale	Studies	Items	Dimensions	Cronbach α	Application time	Sp. val.	Reference
MPQ	Melzack (1975)	78	Sensory, affective, evaluative, miscellaneous	0.70-0.89	15-20 min	Yes	[22]
SF-MPQ	Melzack (1987)	15	Sensory (11) + affective (4) + VAS + PPI	0.73-0.89	5-7 min	Yes	[27]
SF-MPQ-2	Dworkin et al. (2009)	22	Continuous, intermittent, neuropathic, affective	0.83-0.95	5-7 min	Yes	[23,24]
BPI/BPI-SF	Cleeland and Ryan (1994)	32/15	Intensity, location, functional interference	0.82-0.95	5 min	Yes	[25]
WHYMPI	Kerns et al. (1985)	52	Subjective experience, responses, activities	0.70-0.90	15-20 min	Partial	[26]

Neuropathic Pain-Specific Scales

Neuropathic pain, resulting from a lesion or disease of the somatosensory system, possesses distinctive semiological characteristics that justify the development of specific instruments. The DN4, developed under the auspices of the IASP Neuropathic Pain Special Interest Group, combines self-report items (sensations of burning, painful cold, electric shock, tingling, pinprick, numbness, itching) with clinical examination items (tactile hypoesthesia, pinprick hypoesthesia, brush-evoked allodynia) [28]. The original validation in a mixed sample demonstrated excellent discriminative capacity, supported by an elevated DOR and a Youden index close to optimal. However, recent studies in polyneuropathy have shown that its diagnostic performance deteriorates markedly in that context, with a fall in specificity and substantial reduction of DOR [29]. This dependence on clinical context cautions against a critical extrapolation of figures obtained in the original validation.

The Leeds Assessment of Neuropathic Symptoms and Signs (LANSS) combines self-report items with two physical examination components (allodynia, alteration of pinprick threshold) [30]. The self-administered LANSS (S-LANSS) version eliminates examination to facilitate population screening. The painDETECT questionnaire (PDQ), originally developed in Germany for low back pain with a neuropathic component, is entirely self-reported and explores pain quality and intensity, its temporal course, and irradiation pattern [31]. Its score allows three diagnostic categories (unlikely, ambiguous, probable). It has been validated cross-culturally in more than 20 languages, including Spanish. A recent conceptual limitation is that LANSS, DN4, and painDETECT were developed before the introduction of the nociplastic pain concept, the third mechanistic descriptor of IASP (2017), which designates pain by central sensitization without peripheral nerve lesion [6]. Therefore, they do not allow discrimination between neuropathic and nociplastic pain. Patients with fibromyalgia or central sensitization syndromes may score positively on these questionnaires, reducing their specificity. This consideration recommends using these instruments as screening tools, not as definitive diagnostic standard. Table 6 synthesizes the comparative diagnostic performance of principal neuropathic instruments with derived indices according to the methodology.

**Table 6:** Diagnostic performance of neuropathic pain screening scales - derived indices. LR+ interpretation: > 10 strong diagnostic evidence, 5-10 moderate, 2-5 weak but useful. DOR interpretation: > 25 very high discriminative capacity, 10-25 good, < 10 modest. LR+: positive likelihood ratio (sens/{1-spec}); LR-: negative likelihood ratio ({1-sens}/spec); DOR: diagnostic odds ratio (LR+/LR-); Youden J: sens + spec - 1. nr: not reported in the referenced study; nc: not calculable from AUC without paired sens/spec; LANSS: Leeds Assessment of Neuropathic Symptoms and Signs; S-LANSS: self-administered LANSS; AUC: area under the curve

Scale	Studies	Sens (%)	Spec (%)	LR+	LR-	DOR	Youden J	Reference
DN4	Bouhassira et al. (2005) (original validation, n = 160)	83	90	8.30	0.19	43.9	0.73	[28]
DN4	Dunker et al. (2023) (polyneuropathy)	87	≤ 65	2.49	0.20	12.4	0.52	[29]
LANSS	Bennett (2001) (n = 200)	85	80	4.25	0.19	22.7	0.65	[30]
S-LANSS	Ríos-León et al. (2024) (acute whiplash, AUC > 0.85)	nr	nr	nc	nc	nc	nc	[32]
painDETECT	Freynhagen et al. (2006) (n = 8,000)	85	80	4.25	0.19	22.7	0.65	[31]

Pediatric Scales

Pain assessment in pediatrics demands instruments adapted to cognitive-communicative development. Three age groups are distinguished with specific instruments. For neonates and infants, the Premature Infant Pain Profile-Revised (PIPP-R) integrates behavioral parameters (facial expression), physiological (heart rate, oxygen saturation), and contextual (gestational age, alertness state) and constitutes the standard for neonates up to the first month of life [33]. The Neonatal Infant Pain Scale (NIPS) combines behavioral and physiological dimensions, while the Neonatal Pain, Agitation and Sedation Scale (N-PASS) is preferred in neonatal intensive care. For preschoolers and preverbal children (two months to seven years), the Face, Legs, Activity, Cry, Consolability Scale (FLACC) is the most used observational tool, with robust validity and reliability in postoperative, procedural, and disease-related pain [34]. The revised version (FLACC-R) incorporates descriptors adapted to children with cognitive disability. For school-aged children and adolescents (≥ 4 years with verbal capacity), self-report can begin with the FPS-R [20]. From seven to eight years of age, NRS and VAS are applicable with increasing validity. Table 7 synthesizes the selection of pediatric scales by age group.

**Table 7:** Selection of pediatric scales according to age group and respondent type. NICU: neonatal intensive care unit; CHEOPS: Children’s Hospital of Eastern Ontario Pain Scale; PIPP-R: Premature Infant Pain Profile-Revised; NIPS: Neonatal Infant Pain Scale; N-PASS: Neonatal Pain, Agitation and Sedation Scale; FLACC: face, legs, activity, cry, consolability; FLACC-R: FLACC revised version; FPS-R: Faces Pain Scale-Revised; VAS: visual analog scale; NRS: numerical rating scale

Age group	Scale	Type	Domains evaluated	Range	Reference
Premature and term neonates (0-1 month)	PIPP-R	Observational	Facial, physiological, contextual	0-21	[33]
Neonates (NICU)	NIPS	Observational	Behavioral + physiological (6 dimensions)	0-7	[35]
Neonates (NICU)	N-PASS	Observational	Acute/chronic pain + sedation	Variable	[36]
Infants and preschoolers (2 months to 7 years)	FLACC/FLACC-R	Observational	Face, legs, activity, cry, consolability	0-10	[34]
Postsurgical preschoolers	CHEOPS	Observational	Cry, facial, verbal, torso, touch, legs	4-13	[37]
Children ≥ 4 years with verbal capacity	FPS-R	Pictographic self-report	6 faces (neutral)	0-10	[20]
Children ≥ 3 years in emergency	Wong-Baker	Pictographic self-report	6 faces (with affect)	0-10	[21]
Adolescents (≥ 7-8 years)	NRS/VAS	Self-report	Numerical/visual intensity	0-10	[5,17]

Scales for Non-communicative Critically Ill Patients

The critically ill patient under sedation-analgesia or mechanical ventilation poses a particular challenge as follows: the "gold standard" self-report of pain is unfeasible in up to 70% of ICU patients. The Pain, Agitation/Sedation, Delirium, Immobility, Sleep (PADIS) guideline of the Society of Critical Care Medicine recommends as behavioral instruments of choice as follows: the Behavioral Pain Scale (BPS), which evaluates three behavioral domains in intubated patients (facial expression, upper extremity movements, and tolerance or synchrony with the ventilator), with the non-intubated version (BPS-NI) substituting ventilator synchrony for vocalization; and the Critical-Care Pain Observation Tool (CPOT), which adds a fourth domain (muscle tension) to the conceptual structure of the BPS [38-40]. Comparative studies in mechanically ventilated patients show a complementary diagnostic profile as follows: CPOT exhibits greater sensitivity, while BPS contributes greater specificity and, consequently, a superior DOR (Table 8) [41].

**Table 8:** Diagnostic comparison CPOT vs. BPS in critically ill patients. LR+, LR-, DOR, and Youden J calculated per standard formulas; expected combined sensitivity under conditional independence hypothesis. CPOT: Critical-Care Pain Observation Tool; BPS: Behavioral Pain Scale; LR+: positive likelihood ratio; LR-: negative likelihood ratio; DOR: diagnostic odds ratio; nr: not reported

Parameter	CPOT	BPS	Combination
Sensitivity	76.5%	62.7%	80% (observed)/91.2% (expected parallel)
Specificity	70.8%	91.7%	nr
LR+	2.62	7.55	-
LR-	0.33	0.41	-
DOR	7.9	18.6	-
Youden J	0.47	0.54	-
Cutoff	≥ 3/8	≥ 5/12	-
Interrater reliability	κ = 0.80-0.90	κ > 0.80	-

The combination of the two scales elevates the observed sensitivity, suggesting operational complementarity rather than substitution. It is relevant to note that this combined sensitivity is inferior to that expected under the conditional independence assumption between instruments, a finding consistent with the partial overlap of the two in the facial expression domain, which is, moreover, the most responsive to change [41].

Scales for Patients With Cognitive Impairment and Dementia

Pain prevalence in dementia ranges between 60% and 80%, and the deterioration of verbal communication makes recourse to behavioral observational scales indispensable. The American Geriatrics Society and the British Pain Society propose an assessment hierarchy as follows: (1) attempt at self-report adapted to cognitive level (preferably with FPS-R or VRS); (2) structured behavioral observation with a validated scale, at rest and during mobilization; (3) systematic analysis of somatic causes [42]. The Pain Assessment in Advanced Dementia (PAINAD) evaluates five behavioral domains (breathing, negative vocalization, facial expression, body language, consolability) [43]. Its operational brevity and excellent interrater reliability have made it the standard for advanced dementia. The Spanish validation is widely disseminated in geriatric residences. DOLOPLUS-2, of French origin, evaluates items grouped in three dimensions (somatic, psychomotor, and psychosocial) [44]. The Abbey Pain Scale, originating in Australia, evaluates dimensions of vocalization, facial expression, body language changes, and behavioral, physiological, and physical indicators [45]. The Mobilization-Observation-Behavior-Intensity-Dementia Pain Scale (MOBID-2), developed in Norway, evaluates the painful response during guided maneuvers of joint mobilization plus general behavior during care, and is the scale with the greatest evidence in randomized clinical trials for detection of musculoskeletal pain in dementia [46]. Table 9 comparatively summarizes the observational scales for non-communicative populations.

**Table 9:** Observational scales by target population - operational comparison. Cutoff: score above which clinically significant pain is considered per primary literature; Sp. val.: Spanish validation; FLACC: Face, Legs, Activity, Cry, Consolability scale; PIPP-R: Premature Infant Pain Profile-Revised; BPS: Behavioral Pain Scale; BPS-NI: Behavioral Pain Scale non-intubated version; CPOT: Critical-Care Pain Observation Tool; PAINAD: Pain Assessment in Advanced Dementia; MOBID-2: Mobilization-Observation-Behavior-Intensity-Dementia

Scale	Population	Principal domains	Range (cutoff)	Time	Sp. val.	Reference
FLACC	Children 2 months - 7 years	Face, legs, activity, cry, consolability	0-10	1-2 min	Yes	[34]
PIPP-R	Premature and term neonates	Facial, physiological, contextual	0-21	1 min	Partial	[33]
BPS/BPS-NI	ICU adult intubated/non-intubated	Facial, arms, ventilator/vocalization	3-12 (≥ 5)	< 1 min	Yes	[39]
CPOT	ICU adult	Facial, movements, muscle tone, ventilator	0-8 (≥ 3)	< 1 min	Yes	[40]
PAINAD	Advanced dementia	Breathing, vocalization, facial, body, consolability	0-10 (≥ 2)	1-2 min	Yes	[43]
DOLOPLUS-2	Elderly with dementia	Somatic, psychomotor, psychosocial	0-30 (≥ 5)	5 min	Yes	[44]
Abbey	Terminal dementia/palliative	Vocalization, facial, body, behavioral, physiological, physical	0-18	1 min	Partial	[45]
MOBID-2	Dementia + joint mobilization	Response to guided mobilization	0-10	3-5 min	Partial	[46]

Functional and Quality-of-Life Scales

The integral assessment of chronic pain demands instruments that quantify its functional impact and impact on quality of life, dimensions explicitly recommended by IMMPACT [5]. The Oswestry Disability Index (ODI) is the international standard for evaluating functional disability associated with chronic low back pain in spinal pathology, with robust psychometric properties and validated MCID [47]. The Western Ontario and McMaster Universities Osteoarthritis Index (WOMAC) is a specific instrument for hip and knee osteoarthritis, structured into three dimensions (pain, stiffness, physical function), and is the international standard in rheumatological osteoarthritis research [48]. The Short Form Health Survey (SF-36) is the most widely used generic health-related quality-of-life questionnaire worldwide, evaluating eight domains spanning physical, social, emotional, and mental dimensions [49]. The SF-12 version preserves the fundamental properties in reduced format [50]. The Roland-Morris Disability Questionnaire (RMDQ) consists of dichotomous (yes/no) statements that the patient endorses according to how they affect their current day. It is more responsive to change than the ODI in mild-to-moderate low back pain, and its brevity makes it operationally preferable in primary care [51]. Table 10 summarizes the functional and quality-of-life scales.

**Table 10:** Functional and quality-of-life scales in pain - psychometric characteristics. Sp. val.: Spanish validation; MCID: minimal clinically important difference; ODI: Oswestry Disability Index; RMDQ: Roland-Morris Disability Questionnaire; WOMAC: Western Ontario and McMaster Universities Osteoarthritis Index

Scale	Studies	Items	Target population/pathology	Range	MCID	Sp. val.	Reference
ODI	Fairbank and Pynsent (2000)	10	Chronic low back pain	0-100%	6 points (10%)	Yes	[47]
RMDQ	Roland and Morris (1983)	24	Mild-moderate low back pain	0-24	2-5 points	Yes	[51]
WOMAC	Bellamy et al. (1988)	24	Hip/knee osteoarthritis	0-96	12% baseline	Yes	[48]
SF-36	Ware Jr and Sherbourne (1992)	36	Generic quality of life	0-100 (8 domains)	5 points per domain	Yes	[49]
SF-12	Ware Jr et al. (1996)	12	Quality of life (brief)	0-100	3-5 points	Yes	[50]

Psychological and Cognitive Scales of Pain

The biopsychosocial model of pain demands instruments that quantify the cognitive-affective processes modulating the pain experience and therapeutic response. The Pain Catastrophizing Scale (PCS) evaluates pain catastrophizing in the following three subscales: rumination, magnification, and helplessness [52]. This construct consistently predicts pain chronicity, disability, and poor response to surgical interventions [53]. The Tampa Scale of Kinesiophobia (TSK) evaluates fear of movement or (re)injury, a central factor in Vlaeyen's fear-avoidance model, and has validated short versions (TSK-11, TSK-13) [54]. Its intercorrelation with the PCS and with disability confirms the coherence of the avoidance construct; the shared variance fraction with PCS, close to one-third, indicates that catastrophizing and kinesiophobia are distinct but partially overlapping constructs. The Fear-Avoidance Beliefs Questionnaire (FABQ) was developed specifically for low back pain and is structured in two subscales (physical activity and work) [55]. Its work subscale constitutes the most potent single predictor of chronicity and work disability in acute low back pain. The Pain Self-Efficacy Questionnaire (PSEQ) evaluates the patient's belief in their capacity to function despite pain, a key predictor of success in multidisciplinary chronic pain programs [56]. Table 11 synthesizes the psychological instruments.

**Table 11:** Psychological and cognitive scales of pain. All instruments have validated Spanish versions, generally with good internal consistency (Cronbach α > 0.80). Sp. val.: Spanish validation; PCS: Pain Catastrophizing Scale; TSK: Tampa Scale of Kinesiophobia; FABQ: Fear-Avoidance Beliefs Questionnaire; PSEQ: Pain Self-Efficacy Questionnaire

Scale	Studies	Items	Construct evaluated	Subscales	Sp. val.	Reference
PCS	Sullivan et al. (1995)	13	Pain catastrophizing	Rumination, magnification, helplessness	Yes	[52,53]
TSK	Miller et al. (1991)	17/11/13	Kinesiophobia (fear of movement)	Activity avoidance, somatic focus	Yes	[54]
FABQ	Waddell et al. (1993)	16	Fear-avoidance beliefs	Physical activity, work	Yes	[55]
PSEQ	Nicholas (2007)	10	Pain self-efficacy	Unidimensional	Yes	[56]

Specific and Composite Instruments

The Edmonton Symptom Assessment Scale (ESAS) is a multi-symptom instrument evaluating pain, fatigue, drowsiness, nausea, anorexia, dyspnea, depression, anxiety, and well-being [57]. It constitutes the international standard in oncological palliative care. The Defense and Veterans Pain Rating Scale (DVPRS) is a composite scale developed for the military and veterans context, integrating a numerical intensity assessment with pictographic representation of faces, categorical descriptors, and supplementary questions on functional interference [58]. The pain, enjoyment, general activity (PEG) is an ultra-brief instrument derived from the BPI and validated for screening in primary care [59]. Its elevated concordance with the complete BPI and its brevity make it ideal for population implementation. Table 12 synthesizes the composite and screening instruments.

**Table 12:** Specific and composite instruments for particular clinical contexts. r²: variance fraction explained (concordance between brief instruments and their extended reference versions); DVPRS: Defense and Veterans Pain Rating Scale; PEG: pain, enjoyment, general activity; ESAS: Edmonton Symptom Assessment Scale; NRS: numerical rating scale; BPI: Brief Pain Inventory

Scale	Studies	Items	Use context	Characteristics	Reference
ESAS	Bruera et al. (1991)	10	Oncological palliative care	NRS 0-10 multi-symptom	[57]
DVPRS	Polomano et al. (2016)	5 (NRS + 4 functional)	Military/veterans	Composite: intensity + interference	[58]
PEG	Krebs et al. (2009)	3	Primary care (screening)	BPI-derived; r > 0.80 with full BPI (r² > 0.64)	[59]

Principal findings and derived diagnostic indices

The present review critically synthesizes the psychometric evidence for more than 50 pain assessment instruments used in contemporary algological practice, organizing them according to a four-axis operational taxonomy (respondent type, dimensionality, mechanistic descriptor, and target population). The analysis contributes the following three differential elements with respect to previous narrative reviews: the systematic application of the COSMIN-PROM framework to the totality of included instruments, the calculation and clinical interpretation of derived diagnostic indices (positive and negative likelihood ratios, diagnostic odds ratio, Youden index) for neuropathic and critical-care screening scales, and the explicit incorporation of the Ibero-American perspective regarding the availability of culturally adapted versions and regional validation gaps.

The calculation of derived diagnostic indices yields a more clinically useful reading than the mere comparison of point sensitivity and specificity (Table 6). In the original validation of the DN4, a diagnostic odds ratio close to 44 and a Youden index above 0.70 place the instrument in the range of "very high" diagnostic utility. However, this performance is not critically transferable to other clinical contexts as follows: in polyneuropathy, the diagnostic odds ratio collapses to values close to 12 and the Youden index falls below 0.55, reclassifying the DN4 from an instrument of "very high" discriminative capacity in its original context to one of merely "good" capacity in the peripheral neuropathic context [29]. This dependence on clinical context has a direct practical implication as follows: the clinician must evaluate the local prevalence of the target condition before extrapolating the predictive value of a screening instrument. In the domain of pain assessment in critically ill patients, the CPOT vs. BPS comparison illustrates a frequently overlooked phenomenon (Table 8). The observed combined sensitivity when applying both scales in parallel (80%) is substantially lower than the combined sensitivity expected under the conditional independence assumption between instruments (close to 91%). This gap should not be interpreted as a weakness of the combined approach, but as quantitative evidence of partial correlation between both scales, which share the facial expression domain. The clinical implication is that operational CPOT-BPS complementarity is real but limited by the overlap in their observational substrates, and that integrating a third independent domain could increase aggregate sensitivity more efficiently than the simple sum of behavioral instruments.

Contextual selection and the operator factor

It must be assumed that no universally optimal scale exists as follows: selection must be contextual and consider age, cognitive and communicative status, the mechanistic descriptor of pain, evaluation objectives, and operational feasibility. The most frequent clinical error is applying the VAS or NRS to populations for which they were not designed (elderly with moderate cognitive impairment, patients with aphasia, children under seven, non-communicative critically ill patients) without resorting to specific observational instruments for those contexts. This error illustrates a broader and often-neglected source of measurement error - the operator factor. Scale selection is itself a training-dependent diagnostic decision, and the reliability coefficients reported for observational instruments presuppose trained raters; under the framework of generalizability theory, the rater constitutes an irreducible facet of variance that uncontrolled clinical practice frequently ignores. Inadequate operator competence operates bidirectionally toward iatrogenesis as follows: under-detection leading to undertreated pain in non-communicative patients, and misclassification leading to unnecessary analgesic, opioid, or sedative escalation. The epidemiological link between the uncritical "fifth vital sign" mandate and opioid overprescription is, in this sense, a paradigmatic example of operator- and system-level iatrogenesis derived from metric misuse, and reinforces that structured training and competency certification in pain metrics should be regarded as a prerequisite, not an accessory, of valid assessment [4]. Pain assessment should also be multidimensional whenever possible. The still-dominant practice of quantifying intensity exclusively through a unidimensional scale captures a single dimension of the painful phenomenon, the sensory-discriminative one, and neglects its affective-motivational and functional dimensions, which are frequently the most relevant for prognosis, therapeutic choice, and patient satisfaction [5,60]. The IMMPACT approach (integrating intensity, physical function, emotional function, and global perception of improvement) constitutes the methodological standard toward which clinical practice should tend [5,60].

The challenge of nociplastic pain and emerging perspectives

The introduction of the nociplastic pain concept by IASP in 2017 has generated a structural conceptual challenge for traditional neuropathic screening scales [6]. DN4, LANSS, and painDETECT were developed before the formalization of central sensitization as an independent mechanism, and therefore do not allow reliable discrimination between peripheral neuropathic pain and nociplastic pain [61]. Patients with fibromyalgia or central sensitization syndromes may score positively on these questionnaires, a phenomenon that reduces their effective specificity in populations with high prevalence of nociplastic comorbidity. The operational consequence is twofold as follows: these instruments must be used for screening rather than as a definitive diagnostic standard, and there is an urgent need for development or adaptation of specific scales for the nociplastic descriptor, which currently have only preliminary instruments and provisional clinical criteria proposed by Kosek et al. [61]. Beyond these provisional criteria, the instruments currently proposed for the nociplastic domain remain at an early stage of psychometric development. Most capture the broader construct of central sensitization rather than nociplastic pain as formally defined, are anchored in self-reported symptom inventories without an independent reference standard, and have been validated predominantly in single-syndrome samples such as fibromyalgia, which limits their discriminant performance across the heterogeneous populations in which nociplastic mechanisms coexist with nociceptive or neuropathic pain. Their cross-cultural adaptation to Spanish- and Portuguese-speaking populations is, moreover, incomplete. Consequently, no instrument can yet be recommended as a validated standard for nociplastic pain, and the development and COSMIN-compliant validation of mechanism-specific instruments, evaluated against the emerging clinical criteria rather than against neuropathic references, remains an open methodological priority. Artificial intelligence applied to automatic pain detection through facial expression analysis, based on the Facial Action Coding System and on convolutional neural network architectures, represents an emerging horizon with promising preliminary results in non-communicative populations [62]. Its potential integration into continuous monitoring systems in intensive care units, geriatric residences, and pediatric neonatal settings could reshape pain assessment in populations where self-report is infeasible. Nonetheless, the available evidence remains scarce and comes predominantly from monocenter studies with modest sample sizes; before its widespread clinical adoption, prospective multicenter validation per COSMIN standards is required, evaluation of algorithmic equity for demographic biases, and rigorous characterization of false positives in non-painful emotional states. A legitimate objection is whether incorporating such tools would further increase the heterogeneity that already precludes quantitative synthesis in this field. The answer is conditional and relocates rather than amplifies the problem. A trained, version-fixed model behaves as a digitally reproducible rater, collapsing the inter-observer variance that burdens human-scored observational scales and thereby mitigating the operator factor. Conversely, it shifts variance toward model architecture, deployment conditions, and, most critically, demographic performance disparities. The precise psychometric concern is therefore not increased population heterogeneity but a potential failure of measurement invariance across the existing population strata, the same equivariance problem underlying the cultural modulation of pain expression and the Ibero-American cross-cultural adaptation gap. Accordingly, AI does not create a new category of bias but intensifies a pre-existing one. Under standardized, pre-registered, demographically stratified COSMIN-compliant validation with shared reference standards, a version-fixed algorithm could prove more amenable to meta-analytic synthesis than the current mosaic of human-rated scales; absent such governance, the opposite holds.

Cross-cultural adaptation gap in the Ibero-American context

Psychometric properties are not intrinsic attributes of the instrument but products of the interaction among instrument, population, and application context. A scale with excellent validation in an Anglo-Saxon population may have suboptimal psychometric performance in a Spanish-speaking population if it has not been culturally adapted and revalidated per COSMIN standards [12]. The present review identified substantial heterogeneity in the availability of validated versions for Ibero-American populations as follows: while instruments such as VAS, NRS, BPI, PAINAD, and PCS have rigorous validations in several Spanish-speaking and Portuguese-speaking countries, other key instruments (notably MOBID-2, PACSLAC-II, Abbey) present only partial or absent validations in the region. This gap constitutes a translational research priority for the region and should be addressed through Ibero-American multicenter consortia that conduct linguistic adaptation, cultural equivalence, and psychometric revalidation in accordance with the COSMIN methodology.

Strengths and limitations

This review presents several methodological strengths distinguishing it from previous narrative approaches as follows: the prospective character of the search protocol and the simultaneous adherence to SANRA and PRISMA-ScR principles allow substantial reproducibility despite the narrative design; the systematic application of the COSMIN-PROM framework contributes a layer of psychometric rigor habitually absent in algological instrument reviews; the calculation and interpretation of derived diagnostic indices contribute a clinically useful reading; the Ibero-American perspective is genuinely integrated; and the inclusion of French-language literature expands the evidential base regarding Francophone-developed scales (DN4, NPSI, DOLOPLUS-2). The limitations are those inherent to a structured narrative review as follows: literature selection, although prospectively guided per SANRA and PRISMA-ScR, does not possess the exhaustiveness or reproducibility of a formal systematic review with blind screening by two independent reviewers; the clinical, population, and methodological heterogeneity made quantitative synthesis through meta-analysis impossible; some relevant instruments (notably the most recent scales specific to nociplastic pain) have scarce psychometric evidence that does not yet allow a clear recommendation; the operator factor, although discussed as a determinant of measurement validity, was not appraised systematically across the included instruments, since most primary studies report reliability under trained-rater conditions without characterizing performance as a function of operator competence; data extraction was performed by reviewers not blinded to the general review hypothesis, constituting a confirmation bias risk that was mitigated through a priori prioritization of COSMIN-compliant studies; and the temporal coverage up to February 2026 implies that subsequent publications are not included.

Future research directions

Six lines of research emerge as priorities - first, conducting systematic reviews with population-stratified meta-analyses per COSMIN methodology; second, development or adaptation of specific instruments for the nociplastic mechanistic descriptor; third, generation of responsiveness studies with prolonged follow-up for observational geriatric scales; fourth, conformation of Ibero-American multicenter consortia for cross-cultural adaptation and revalidation; fifth, prospective multicenter validation of emerging artificial intelligence-based pain detection systems, with specific evaluation of algorithmic equity and of false positives in non-painful emotional states; and sixth, the design of studies quantifying the operator-dependent component of observational pain assessment through generalizability-theory designs, alongside the evaluation of structured training and competency-certification programs in pain metrics.

## Conclusions

No universal pain assessment scale exists - optimal selection depends on clinical context, age, cognitive status, the mechanistic descriptor of pain, and the evaluation objectives. In cognitively intact adults, the NRS constitutes the IMMPACT standard for intensity and the BPI for functional interference, while the SF-MPQ-2 adds the qualitative characterization of neuropathic and nociceptive components; in non-communicative populations, specific observational tools (FLACC and PIPP-R in preverbal pediatrics, BPS and CPOT in intensive care, PAINAD, DOLOPLUS-2, and MOBID-2 in advanced dementia) are irreplaceable. As discussed, neuropathic screening scales retain clinical utility but do not reliably discriminate nociplastic pain and should therefore be understood as orientation tools rather than definitive diagnostic standards. The complementary integration of functional, quality-of-life, cognitive, and behavioral instruments operationalizes the biopsychosocial approach recommended by international guidelines and should be understood as the contemporary standard for comprehensive assessment of chronic pain. The aggregated methodological quality of the body of evidence is globally high, but specific areas warrant priority research, as follows: the scarcity of responsiveness studies for observational geriatric scales, the limited discriminant validity of neuropathic vs. nociplastic screening, and the heterogeneity of cross-cultural adaptation to Spanish and Portuguese. Addressing the latter through COSMIN-compliant revalidation of Ibero-American versions, ideally via multicenter consortia, remains a translational priority for the region. Emerging perspectives based on artificial intelligence and continuous pain monitoring in non-communicative populations open a promising horizon but demand prospective multicenter validation and evaluation of algorithmic equity before generalized clinical adoption. In this scenario, no scale, however sophisticated, can substitute for integrative clinical judgment; the valid quantitative tool and the professional's clinical sensitivity together constitute the optimal standard of contemporary algological practice.
